# Evidence of avian influenza virus in seabirds breeding on a Norwegian high-Arctic archipelago

**DOI:** 10.1186/s12917-020-2265-2

**Published:** 2020-02-07

**Authors:** Megan Marie Lee, Veerle L. B. Jaspers, Geir Wing Gabrielsen, Bjørn Munro Jenssen, Tomasz Maciej Ciesielski, Åse-Karen Mortensen, Silje Strand Lundgren, Courtney A. Waugh

**Affiliations:** 1 Department of Biology, Norwegian University of Science and Technology, Høgskoleringen 5, NO-7491 Trondheim, Norway; 2 Biological Sciences Program, Goucher College, 1021 Dulaney Valley Road, Baltimore, MD 21204 USA; 3 Norwegian Polar Institute, Fram Centre, Postbox 6606 Langnes, NO-9296 Tromsø, Norway; 4 Faculty of Biosciences and Aquaculture, Nord University, Steinkjer, Trøndelag Norway

**Keywords:** Arctic, Avian influenza, Svalbard, Kittiwake, Glaucous gull

## Abstract

**Background:**

Wild aquatic birds serve as the natural reservoir for avian influenza virus (AIV), a disease with significant implications for avian and mammalian health. Climate change is predicted to impact the dynamics of AIV, particularly in areas such as the Arctic, but the baseline data needed to detect these shifts is often unavailable. In this study, plasma from two species of gulls breeding on the high-Arctic Svalbard archipelago were screened for antibodies to AIV.

**Results:**

AIV antibodies were found in black-legged kittiwake *(Rissa tridactyla)* samples from multiple years, as well as in glaucous gulls *(Larus hyperboreous)* samples.

**Conclusions:**

Despite small sample sizes, evidence of exposure to AIV was found among Svalbard gulls. A wider survey of Svalbard avian species is warranted to establish knowledge on the extent of AIV exposure on Svalbard and to determine whether active infections are present.

## Background

Avian influenza viruses (AIVs) are adapted to avian species, and aquatic birds are the natural hosts and reservoir species, as a consequence of that there is extensive genetic diversity in aquatic birds [1]. AIVs in wild birds tend to have a low pathogeneicity when it comes to overt clinical signs [2]. AIVs from wild birds can elicit lethal symptoms when transmitted to non-reservoir species, for example causing mass die offs in marine mammals [3]. Additionally, AIV is an important zoonotic disease and increased circulation of the virus may facilitate the emergence of strains that can pass from wild birds to domestic poultry and to humans [4]. Thus, AIV has the potential to significantly impact human and wildlife health on a global scale.

Dynamics of AIV may be particularly affected in heavily climate-stressed areas, such as the Arctic [4–6]. Since 1980, the annual temperature increase has been twice as high in the Arctic as in the rest of the world [7]. Novel shifts in Arctic disease dynamics are already documented and the effects are predicted to amplify due to these warming trends [7]. Baseline data in the Arctic on the presence and prevalence of pathogens in wildlife is therefore critical for predicting and tracking further shifts [7]. Svalbard.

While ducks and geese have the highest infection rates among wild birds [1], gulls also form a significant component of the AIV reservoir. The viral subtypes that dominate in gulls and shorebirds are not often found in ducks and geese [8], suggesting that these species may harbor a separate pool of AIV genetic diversity [1] and might thereby increase the potential for the emergence of new reassortant strains. Northern breeding grounds have been implicated as sites of high AIV density in North America [2]. Such sites, where birds from disparate populations congregate, may be important areas of AIV transmission [8].

This investigation aimed to serve as a pilot study on the presence of AIV antibodies in breeding populations of seabirds in an Arctic area, namely Svalbard, Norway. The presence of AIV antibodies was determined in the plasma of two species of Arctic gulls: black-legged kittiwakes (Rissa tridacyla) and glaucous gulls (*Larus hyperboreus*) from breeding colonies in Kongsfjorden. The black-legged kittiwake is a medium-sized gull with highly pelagic habits. Its circumpolar distribution and status as the most numerous gull in the world makes it a good respresentative species for Arctic seabirds. During the breeding season, kittiwakes nest in dense colonies, which can consist of tens of thousands of individuals. On Svalbard, they may share ecological interactions with other bird and mammal species during this time. Eggs, chicks, and occasional adults are preyed upon by the Arctic fox *(Vulpes lagopus),* glaucous gull*,* great skua *(Stercorarius skua),* and Arctic skua *(Stercorarius parasiticus),* while Brünnich’s guillemots *(Uria lomvia)* are known to nest in mixed colonies alongside kittiwakes. The glaucous gull has a circumpolar distribution and is one of the largest avian predators in the Arctic. Estimations of the breeding population in Svalbard are between 4000 and 10,000 pairs, and they breed in small colonies or single pairs usually close to colonies of other seabirds. They winter mainly in the North Atlantic Ocean and stay there from around November to March. The glaucous gull has an apex position in the arctic food web and is an opportunistic scavenger, with a diet that varies from pelagic and marine invertebrates, fish, eggs, chicks and adults of other seabirds to carrion or humane refuse. The food preference depends on their breeding ground.

## Results

No antibodies were found in black-legged kittiwakes from 2017, but 7 out of 25 (28%) of the 2015 kittiwakes and 2 out of 16 (12.5%) of the 2014 kittiwakes tested positive, while AIV antibodies were found in 5 of the 15 (33%) glaucous gull samples in 2017 (Table 1). Positive results were found in both male and female birds, and at both kittiwake breeding colonies. The results are determined as the ratio of ELISA optical densities for the specimen and the negative control (S/N), and the S/N ratios ae provided in the Additional files 1 and 2.

**Table 1:** Prevalence of avian influenza virus (AIV) antibodies in the plasma of two seabird species sampled while breeding on Svalbard, Norway between 2014 and 2017. Seabird species sampled were black-legged kittiwakes (*Rissa tridactyla*) and glaucous gulls (*Laurus hyperboreus*)

Species	Year	Location	*n*	Antibody prevalence (%)
Black-Legged Kittiwake	2017	Kongsfjorden	12	0%
Black-Legged Kittiwake	2015	Kongsfjorden	25	28%
Black-Legged Kittiwake	2014	Kongsfjorden	16	12.5%
Glaucous Gull	2017	Adventfjorden/Sassendalen	15	33%

## Discussion

With such small sample sizes, these positive findings are especially notable and warrant a larger epidemiological survey of Svalbardian seabirds and other fauna. While AIV has been previously detected in seabirds from other regions of the Arctic, including black-legged kittiwakes breeding in an Arctic region of mainland Norway [9] and glaucous gulls on an Alaskan island [10], this is, to our knowledge, the first data showing that Svalbardian fauna has been exposed to AIV. Evidence from other regions suggests that AIV exposure on Svalbard may not be limited to the gull species tested here. On one Alaskan island, AIV was detected in seven different species of migratory birds, including common guillemots (*Uria aalge*), Brünnich’s guillemots (*U. lomvia*), and king eiders (*Somateria spectabilis*) as well as glaucous gulls [10]. These four species are all common breeders on Svalbard.

**Fig. 1:**
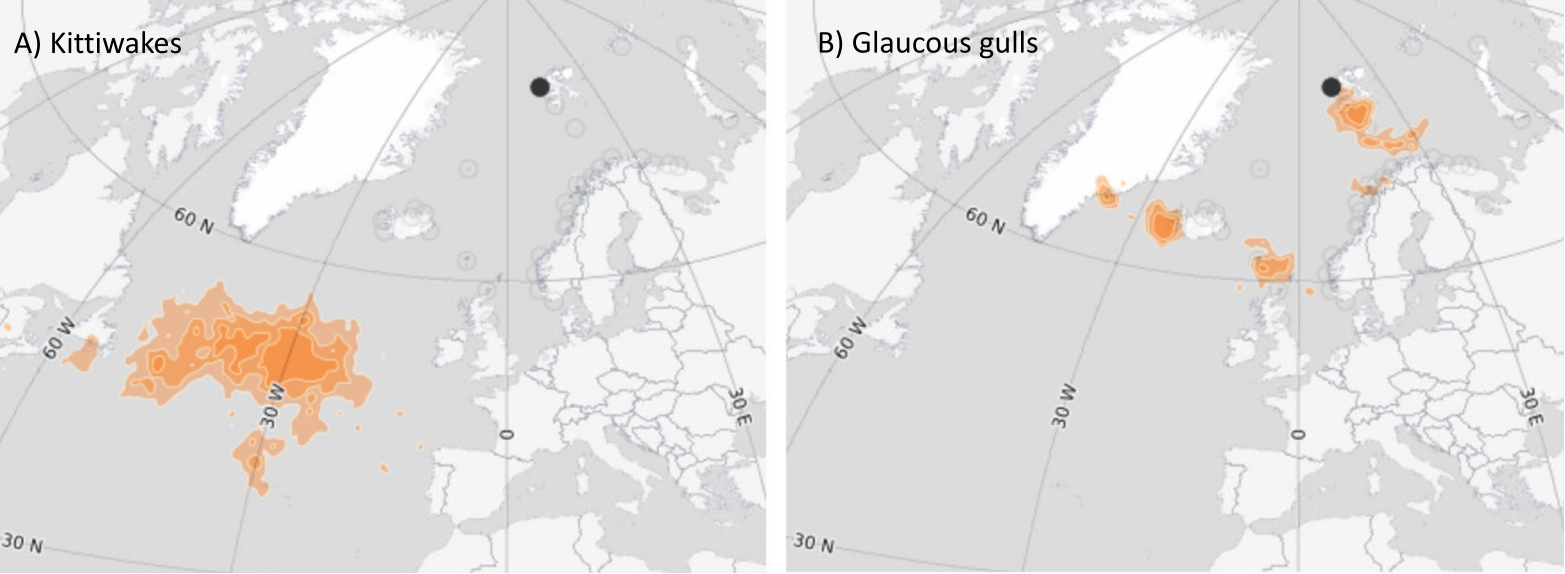
Overwintering areas (organge) of **a**) black-legged kittiwakes (*n* = 22) (*Rissa tridactyla*) and **b**) glaucous gulls (*n* = 6) (*Laurus hyperboreus*). Data extracted from SEATRACK Seabird Tracking http://www.seapop.no/en/seatrack/. Black dot represents their Svalbardian breeding sites

Kittiwakes and glaucous gulls inhabiting Svalbard breeding grounds are both migratory species. Kittiwakes migrate longer distances from Svalbard to their North Atlantic Ocean overwintering grounds (Fig. 1a), whereas glaucous gulls prefer to overwinter in closer proximity south of Svalbard in the Barents Sea, Norwegian Sea and Greenland Sea (Fig. 1b) (Norwegian SEATRACK project; http://www.seapop.no/en/seatrack/). Developing more reliable estimates of AIV prevalence in Svalbard and on their over-wintering grounds, is essential for detecting possible disease shifts in the area and increased risks to humans and wildlife. This study only screened for antibodies and cannot confirm that any birds had an active infection during their time on Svalbard. Using genetic techniques to confirm the presence of the virus and potentially identify the carried strain would be a logical next step.

## Conclusions

This study presents the first evidence of AIV in black-legged kittiwakes and glaucous gulls on Svalbard. Due to this detection we recommend that a wider screening of Svalbard wildlife should be undertaken. Knowledge on AIV ecology in Svalbard is lacking and could have significant implications for avian, mammalian, and human health.

## Methods

Samples were taken between 2014 and 2017 from seabird breeding sites and surrounding areas on the Svalbard archipelago in the Norwegian Arctic. Adult kittiwakes were sampled in 2014 (*n* = 16) and 2015 (*n* = 25) across two breeding colonies (Blomstrandhalvøya and Krykkjefjellet), while in 2017 (*n* = 12) they were sampled at Blomstrandhalvøya only (Additional file 1: Table S1). Kittiwakes were captured on their nests with a snare on a long fishing rod via a well-established method by experienced and well-trained personnel only. No adverse effects were noted due to sampling.

Between 0.1 mL to 2 mL of blood was collected [11].

Adult glaucous gull samples (*n* = 15) were collected during the pre-breeding period in 2017 from locations around Sassendalen and Adventfjorden on Svalbard. Both males (*n* = 7) and females (*n* = 8) were sampled. With ethical permissions from the governor of Svalbard, birds were euthanized by shotgun and followed by decapitation for use in a separate project. Blood was collected either from the neck after decapitation with a heparinized knife or from the heart using a heparinized syringe. Within 30 min, samples were centrifuged for 10 min at 5000 rpm. Plasma aliquots of 50 μL were taken for this study.

Plasma aliquots were screened for AIV antibodies using an Influenza A virus antibody test kit (IDEXX Influenza A kit) based on the ELISA principle. Negative and positive control sera were used in each experiment (provided with the kit). The results are determined as the ratio of ELISA optical densities from the specimen and the negative control (S/N). The manufacturers recommended cutoff of less than or equal to 0.5 for a positive reading was used. Absorbance values were visualized using a Cytation 5 Imaging Reader (BioTek). Percent prevalence of the AIV antibodies was calculated for each sample set.

## Supplementary information

**Additional file 1: Table S1.** Sexes and breeding locations of black-legged kittiwakes *(Rissa tridactyla)* sampled from Kongsfjorden, Svalbard in 3 years. Sexes and breeding locations of black-legged kittiwakes *(Rissa tridactyla)* sampled from Kongsfjorden, Svalbard in 3 years.

**Additional file 2: Table S2.** a) Adult glaucous gull samples (*n* = 15) were collected in 2017 on Svalbard and tested for Avian Influenza antibodies based on the ELISA principle. S/N ratios (the ratio of ELISA optical densities from the specimen and the negative control) are provided. The manufacturers recommended cut-off of less than or equal to 0.5 for a positive reading was used. Negative is ≥0.50; Positive is < 0.50.; b) Adult black-legged kittiwake (*Rissa tridactyla*) samples (*n* = 25) were collected in 2015 on Svalbard and tested for Avian Influenza antibodies based on the ELISA principle. S/N ratios (the ratio of ELISA optical densities from the specimen and the negative control) are provided. The manufacturers recommended cut-off of less than or equal to 0.5 for a positive reading was used. Negative is ≥0.50; Positive is < 0.50.; c) Adult black-legged kittiwake (*Rissa tridactyla*) samples (*n* = 16) were collected in 2014 on Svalbard and tested for Avian Influenza antibodies based on the ELISA principle. S/N ratios (the ratio of ELISA optical densities from the specimen and the negative control) are provided. The manufacturers recommended cut-off of less than or equal to 0.5 for a positive reading was used. Negative is ≥0.50; Positive is < 0.50.; d) Adult black-legged kittiwake (*Rissa tridactyla*) samples (*n* = 12) were collected in 2017 on Svalbard and tested for Avian Influenza antibodies based on the ELISA principle. S/N ratios (the ratio of ELISA optical densities from the specimen and the negative control) are provided. The manufacturers recommended cut-off of less than or equal to 0.5 for a positive reading was used. Negative is ≥0.50; Positive is < 0.50. Raw data for the ELISA results.

## Data Availability

The datasets used and/or analysed during the current study available from the corresponding author on reasonable request.

## Abbreviations

AIV: Avian influenza virus
ELISA: Enzyme-linked immunosorbent assay
LPAI: Low-pathogenic avian influenza
