# Supplementary material for: Evidence of avian influenza virus in seabirds breeding on a Norwegian high-Arctic archipelago
Source: BMC Vet Res. 2020 Feb 7;16:48. doi: 10.1186/s12917-020-2265-2 (PMC7006154; doi:10.1186/s12917-020-2265-2)
Supplement: Supplementary file 1 — Additional file 1: Table S1. Sexes and breeding locations of black-legged kittiwakes (Rissa tridactyla) sampled from Kongsfjorden, Svalbard in 3 years. Sexes and breeding locations of black-legged kittiwakes (Rissa tridactyla) sampled from Kongsfjorden, Svalbard in 3 years. [file 12917_2020_2265_MOESM1_ESM.docx]

Table S1. Sexes and breeding locations of black-legged kittiwakes *(Rissa tridactyla)* sampled from Kongsfjorden, Svalbard in three years.

| **Year** | **Breeding Colony** | | **Sex** |
| --- | --- | --- | --- |
|  | *Blomstrandhalvøya* | *Krykkjefjellet* |  |
| 2014  (*n*=16) | 3 | 3 | F |
|  | 5 | 5 | M |
| 2015  (*n*=25) | 14 | 11 | F |
|  | 0 | 0 | M |
| 2017  (*n*=12) | 6 | 0 | F |
|  | 6 | 0 | M |
