# Supplementary material for: Evidence of avian influenza virus in seabirds breeding on a Norwegian high-Arctic archipelago
Source: BMC Vet Res. 2020 Feb 7;16:48. doi: 10.1186/s12917-020-2265-2 (PMC7006154; doi:10.1186/s12917-020-2265-2)
Supplement: Supplementary file 2 — Additional file 2: Table S2. a) Adult glaucous gull samples (n = 15) were collected in 2017 on Svalbard and tested for Avian Influenza antibodies based on the ELISA principle. S/N ratios (the ratio of ELISA optical densities from the specimen and the negative control) are provided. The manufacturers recommended cut-off of less than or equal to 0.5 for a positive reading was used. Negative is ≥0.50; Positive is < 0.50.; b) Adult black-legged kittiwake (Rissa tridactyla) samples (n = 25) were collected in 2015 on Svalbard and tested for Avian Influenza antibodies based on the ELISA principle. S/N ratios (the ratio of ELISA optical densities from the specimen and the negative control) are provided. The manufacturers recommended cut-off of less than or equal to 0.5 for a positive reading was used. Negative is ≥0.50; Positive is < 0.50.; c) Adult black-legged kittiwake (Rissa tridactyla) samples (n = 16) were collected in 2014 on Svalbard and tested for Avian Influenza antibodies based on the ELISA principle. S/N ratios (the ratio of ELISA optical densities from the specimen and the negative control) are provided. The manufacturers recommended cut-off of less than or equal to 0.5 for a positive reading was used. Negative is ≥0.50; Positive is < 0.50.; d) Adult black-legged kittiwake (Rissa tridactyla) samples (n = 12) were collected in 2017 on Svalbard and tested for Avian Influenza antibodies based on the ELISA principle. S/N ratios (the ratio of ELISA optical densities from the specimen and the negative control) are provided. The manufacturers recommended cut-off of less than or equal to 0.5 for a positive reading was used. Negative is ≥0.50; Positive is < 0.50. Raw data for the ELISA results. [file 12917_2020_2265_MOESM2_ESM.docx]

Table S2a. Adult glaucous gull samples (*n*=15) were collected in 2017 on Svalbard and tested for Avian Influenza antibodies based on the ELISA principle. S/N ratios (the ratio of ELISA optical densities from the specimen and the negative control) are provided. The manufacturers recommended cut-off of less than or equal to 0.5 for a positive reading was used. Negative is ≥ 0.50; Positive is <0.50.

| **S/N** | **Result** |
| --- | --- |
| 1.18 | Negative |
| 0.73 | Negative |
| 0.68 | Negative |
| 0.63 | Negative |
| 0.52 | Negative |
| 0.36 | Positive |
| 0.74 | Negative |
| 0.49 | Positive |
| 0.73 | Negative |
| 0.66 | Negative |
| 0.41 | Positive |
| 0.73 | Negative |
| 0.45 | Positive |
| 0.52 | Negative |
| 0.16 | Positive |

Table S2b. Adult black-legged kittiwake (*Rissa tridactyla*) samples (n = 25) were collected in 2015 on Svalbard and tested for Avian Influenza antibodies based on the ELISA principle. S/N ratios (the ratio of ELISA optical densities from the specimen and the negative control) are provided. The manufacturers recommended cut-off of less than or equal to 0.5 for a positive reading was used. Negative is ≥ 0.50; Positive is <0.50.

| **S/N** | **Result** |
| --- | --- |
| 0.52 | Negative |
| 0.98 | Negative |
| 1.05 | Negative |
| 0.15 | Positive |
| 0.97 | Negative |
| 0.46 | Positive |
| 0.79 | Negative |
| 0.91 | Negative |
| 0.75 | Negative |
| 1.12 | Negative |
| 0.17 | Positive |
| 1.14 | Negative |
| 0.36 | Positive |
| 0.86 | Negative |
| 0.24 | Positive |
| 0.83 | Negative |
| 0.88 | Negative |
| 0.82 | Negative |
| 1.05 | Negative |
| 0.82 | Negative |
| 0.75 | Negative |
| 0.26 | Positive |
| 0.98 | Negative |
| 0.31 | Positive |
| 0.85 | Negative |

Table S2c. Adult black-legged kittiwake (*Rissa tridactyla*) samples (n = 16) were collected in 2014 on Svalbard and tested for Avian Influenza antibodies based on the ELISA principle. S/N ratios (the ratio of ELISA optical densities from the specimen and the negative control) are provided. The manufacturers recommended cut-off of less than or equal to 0.5 for a positive reading was used. Negative is ≥ 0.50; Positive is <0.50.

| **S/N** | **Result** |
| --- | --- |
| 0.09 | Positive |
| 0.70 | Negative |
| 1.27 | Negative |
| 0.92 | Negative |
| 0.79 | Negative |
| 1.24 | Negative |
| 1.01 | Negative |
| 0.91 | Negative |
| 0.97 | Negative |
| 1.17 | Negative |
| 1.22 | Negative |
| 1.04 | Negative |
| 0.94 | Negative |
| 0.53 | Negative |
| 0.24 | Positive |
| 1.15 | Negative |

Table S2d. Adult black-legged kittiwake (*Rissa tridactyla*) samples (n = 12) were collected in 2017 on Svalbard and tested for Avian Influenza antibodies based on the ELISA principle. S/N ratios (the ratio of ELISA optical densities from the specimen and the negative control) are provided. The manufacturers recommended cut-off of less than or equal to 0.5 for a positive reading was used. Negative is ≥ 0.50; Positive is <0.50.

| **S/N** | **Result** |
| --- | --- |
| 1.15 | Negative |
| 1.10 | Negative |
| 1.07 | Negative |
| 1.18 | Negative |
| 1.09 | Negative |
| 0.94 | Negative |
| 0.85 | Negative |
| 1.38 | Negative |
| 1.17 | Negative |
| 1.11 | Negative |
| 1.30 | Negative |
| 1.38 | Negative |
